# HomeStyles, A Web-Based Childhood Obesity Prevention Program for Families With Preschool Children: Protocol for a Randomized Controlled Trial

**DOI:** 10.2196/resprot.7544

**Published:** 2017-04-25

**Authors:** Carol Byrd-Bredbenner, Jennifer Martin-Biggers, Mallory Koenings, Virginia Quick, Nobuko Hongu, John Worobey

**Affiliations:** ^1^ Rutgers University Department of Nutritional Sciences New Brunswick, NJ United States; ^2^ University of Arizona Department of Nutritional Sciences Tucson, AZ United States

**Keywords:** childhood obesity, nutrition, physical activity, sleep, prevention, parents, children

## Abstract

**Background:**

The home environment is where young children spend most of their time, and is critically important to supporting behaviors that promote health and prevent obesity. However, the home environment and lifestyle patterns remain understudied, and few interventions have investigated parent-led makeovers designed to create home environments that are supportive of optimal child health and healthy child weights.

**Objective:**

The aim of the HomeStyles randomized controlled trial (RCT) is to determine whether the Web-based HomeStyles intervention enables and motivates parents to shape the weight-related aspects of their home environments and lifestyle behavioral practices (diet, exercise, and sleep) to be more supportive of their preschool children’s optimal health and weight.

**Methods:**

A rigorous RCT utilizing an experimental group and an attention control group, receiving a bona fide contemporaneous treatment equal in nonspecific treatment effects and differing only in subject matter content, will test the effect of HomeStyles on a diverse sample of families with preschool children. This intervention is based on social cognitive theory and uses a social ecological framework, and will assess: intrapersonal characteristics (dietary intake, physical activity level, and sleep) of parents and children; family interpersonal or social characteristics related to diet, physical activity, media use, and parental values and self-efficacy for obesity-preventive practices; and home environment food availability, physical activity space and supports in and near the home, and media availability and controls in the home.

**Results:**

Enrollment for this study has been completed and statistical data analyses are currently underway.

**Conclusions:**

This paper describes the HomeStyles intervention with regards to: rationale, the intervention’s logic model, sample eligibility criteria and recruitment, experimental group and attention control intervention content, study design, instruments, data management, and planned analyses.

## Introduction

Evidence-based educational materials regarding obesity prevention for parents of preschool children remain scarce. The home environment is where young children spend most of their time and is critically important to supporting behaviors that promote health and prevent obesity. However, the home environment and lifestyle patterns remain understudied, and few interventions have investigated parent-led multifactorial makeovers designed to create home environments that are supportive of optimal child health and healthy child weights [1-7]. Results of studies indicate that home makeovers hold great promise for ameliorating childhood obesity [8-10]. Building upon previous work, HomeStyles was developed to help parents of young children shape or *makeover* (ie, change) their home environment and lifestyles to prevent childhood obesity. HomeStyles has two delivery modes: (1) independent online learning, and (2) in-home, face-to-face, individualized learning facilitated by trained home visitation staff. This paper reports the design and methods for the online delivery mode.

The HomeStyles project was funded by the National Institute of Food and Agriculture, United States Department of Agriculture (award number 2011-68001-30170). This project builds on the parent-directed home kitchen organization/food management makeover proof-of-concept *Shaping Up America’s Kitchen* intervention that was successfully pilot tested with mothers of young children [9,10]. The pilot test findings supported the work of others indicating that teaching adults how to make home environment and lifestyle changes, like those proposed in this project, and building their self-efficacy can have a positive impact on their home environment and health [11-14]. Formative evaluation findings from *Shaping Up America’s Kitchen* revealed that participants rated the intervention’s materials highly (4-page *factivity* [facts + activity] folios) with regards to readability, completeness, relevance, and usefulness [10]. Pilot-test data showed that participant recruitment, retention, and satisfaction were excellent, and that participants significantly improved their health knowledge and self-efficacy [9].

The HomeStyles intervention is intended to enable and motivate parents of preschoolers to shape their home environment and lifestyle behavioral practices to create and support optimal child growth, health, and weights. The intervention is based on Social Cognitive Theory [5] and uses a social ecological framework [15]. HomeStyles targets the home because this environment plays a dominant role in the development of childhood eating and physical activity patterns, and these patterns track across the growing years into adulthood [16-18]. The program targets parents because they are children’s role models, family food gatekeepers, and create the structure/lifestyle environment within the home, and thus strongly influence the obesity-prevention behaviors of children during the growing years [5,16-36]. In addition, parents need more opportunities to gain relevant, practical, nonjudgmental obesity prevention information that is easily implemented in their homes and hectic lifestyles [24]. HomeStyles uses a multifactorial approach because diet, physical activity, and sleep are well-known in the literature to be associated with childhood obesity risk, and the most successful results are likely to be generated by addressing multiple lifestyle practices in a family context [13,37-41]. An online delivery mode was selected because the vast majority of families in the United States have access to the Internet [42], it is a cost-effective delivery method, and it offers an excellent probability of contributing to the sustained availability of project materials after the study ends.

HomeStyles is innovative in that it is family-focused and based on parent-defined quality of life characteristics. Additionally, obesity prevention programs for young children continue to be limited and few obesity prevention programs for any age groups take a multifactorial approach that incorporates a broad array of factors associated with obesity risk that parents can address quickly, easily, and at low (or no) cost in the home environment. HomeStyles also focuses on the home environment, which is critically important to promoting health but remains sorely understudied. Finally, despite their promise for mitigating childhood obesity, few interventions have examined the efficacy of parent-led home environment restructuring that aims to shape these environments to be more supportive of optimal child health. Thus, the aim of the randomized controlled trial (RCT) is to determine whether the Web-based HomeStyles intervention enables and motivates parents in the experimental group to shape the weight-related aspects of their home environments and lifestyle behavioral practices (diet, exercise, and sleep) to be more supportive of their preschool children’s optimal health and weight, compared to those in the control condition.

## Methods

The study protocol involved two groups (experimental and attention control), and was approved by the institutional review boards at the authors’ universities. All participants gave informed consent online before participating in any component of this study.

### Logic Model

**Figure 1:**
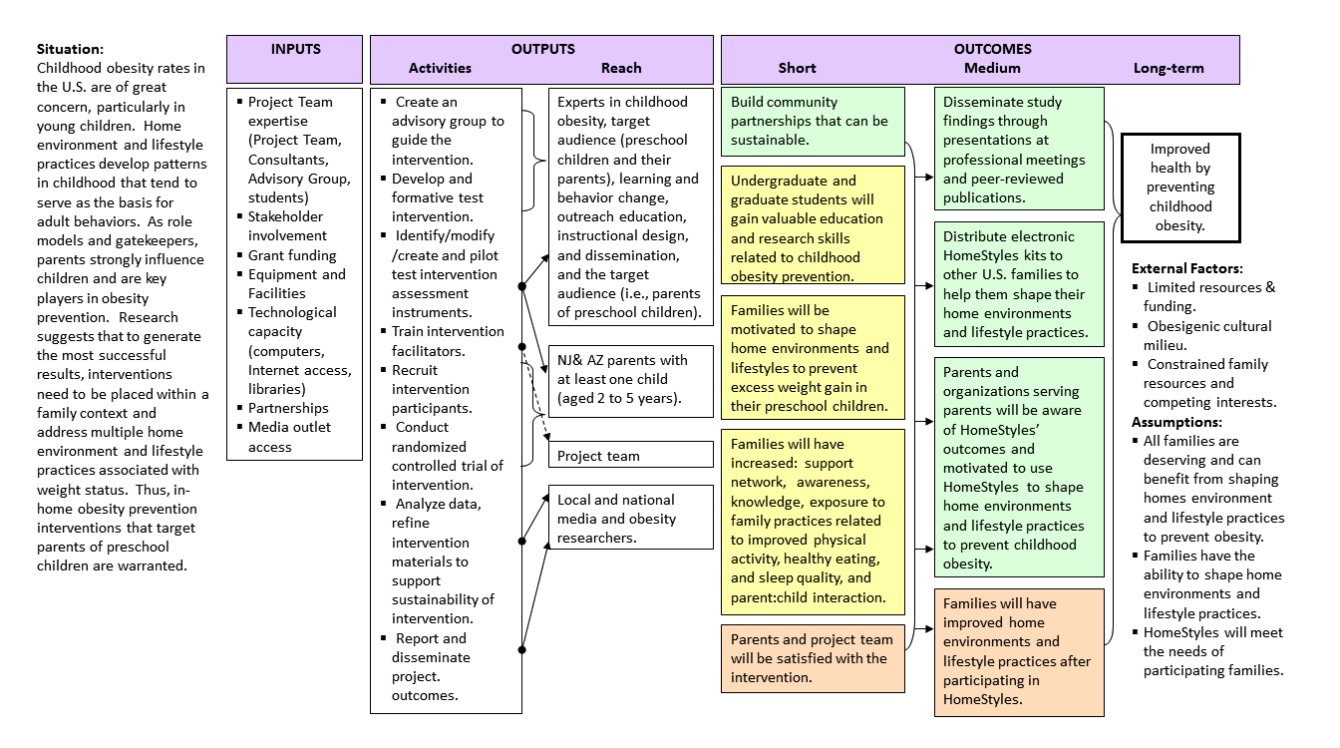
Logic Model for HomeStyles Online Intervention.

As shown in the HomeStyles logic model (Figure 1), the long-term outcome of HomeStyles is to improve health by preventing childhood obesity. To reach the long-term outcome, necessary inputs included: expertise from the project team, consultants, and advisory group; stakeholder involvement at all stages of the project; sufficient funding; equipment, facilities, and technological capacity to complete project development, implementation, and evaluation; partnerships with community leaders and organizations; and access to media to promote HomeStyles.

Eight main activities were needed to reach the project outcomes. The first was to create an advisory group comprised of experts in childhood obesity subject matter (eg, nutrition, exercise/fitness, sleep, child development), the target audience (eg, young children, pediatrics, parenting, social work, cultural competence), learning and behavior change (eg, psychology, behavioral scientists, motivational interviewing, adult learners), outreach education (eg, Cooperative Extension, informal education), instructional design (eg, education, graphic arts, computer technology), and research and outreach dissemination (eg, media, public relations). The second step was to develop and formatively test the HomeStyles intervention, which engaged all experts and target audience members at every step; for details, see the *Intervention* section that follows. The third step was to select, adapt, or create intervention assessment instruments, which are also described in detail in the *Instruments* section. These instruments assess changes that occur while participating in the HomeStyles intervention. The fourth step was to train staff to facilitate the implementation of the intervention and RCT, which is critical to ensuring that all aspects of the intervention are conducted by project staff with uniformity and fidelity to the plan. Activities associated with the fifth step are described in the *Sample Eligibility and Recruitment* section that follows. The final steps were to conduct the RCT to assess the study aims, analyze RCT data, refine intervention materials to improve their quality and acceptability to the target audience, and report and disseminate the project outcomes.

These steps are expected to yield the short-term outcomes of: building sustainable community partnerships; training the next generation of health and nutrition professionals to develop and implement obesity prevention programs; enabling families to shape home environments and lifestyle practices to be preventative of childhood obesity; helping families develop the cognitions, behaviors, and relationships supportive of healthy child weights; and creating a childhood obesity prevention program that is satisfying to parents and those implementing the program. In the medium-term, project outcomes planned are to: disseminate RCT findings to professional groups, widely distribute HomeStyles materials to families, make parents and organizations serving parents aware of HomeStyles and promote its use and adoption, and improve families’ home environments and lifestyle practices. These short- and medium-term outcomes will contribute to achieving the long-term societal outcome of improved health by preventing childhood obesity.

External factors affecting outcomes include: limits on resources and funding; the obesogenic nature of the prevailing culture in the United States; and resource (eg, time, energy, money) scarcity experienced by families, which may impede their progress. HomeStyles is based on the premises that all families are deserving of the opportunity to benefit from shaping home environments and lifestyle practices, all have the capacity to shape home environments and lifestyles, and that HomeStyles will meet their needs. Thus, efforts were made to include families from a variety of sociodemographic backgrounds and geographic locations during program creation and testing.

### Sample Eligibility Criteria and Recruitment

To be eligible for the HomeStyles project, individuals required basic reading and writing skills (either English or Spanish) and had to be parents aged 20 to 45 years. Participants also had to be the household primary food gatekeeper, live in the catchment area (New Jersey and Arizona), have at least one young child (aged 2 to 5 years), and have regular Internet access.

Recruitment materials invited parents of preschool children to participate in a program designed to help them raise, “even happier, healthier, safer children” and directed them to a website to learn more and complete a screener to determine whether they met eligibility requirements. Recruitment materials (eg, printed flyers, posters, bookmarks, and brief videos) were posted to: email listservs of workplaces; philanthropic, religious, and community groups; preschool/day care centers; professional associations; and extracurricular activity/afterschool groups. These materials also were posted to websites (eg, online community newspapers, local businesses, parent blogs), distributed via social media (eg, Facebook, Pinterest), and included in a variety of media (eg, magazines, newspapers, radio, and television). Community partners (eg, pediatric and dietetic associations, fitness centers, schools) as well as personal contacts (eg, colleagues, family, friends, neighbors) and a professional study recruitment company also distributed recruitment materials. In-person recruitment strategies included tabling activities at: community events; parent resource centers; Women, Infants, and Children program offices; and farmers’ markets.

The goal of HomeStyles is obesity *prevention*. However, formative testing revealed that parents strongly disliked the term *obesity* and identified *happier, more closely bonded families* as the key component of a high quality of life and health. These findings drove the thrust and tone of the recruitment materials. Recruitment materials included a link to a webpage that described the study components and expectations, and invited parents to complete a brief questionnaire to determine eligibility.

Recruitment of participants occurred over a 15-month period. The rolling enrollment allowed families to enter at different times of the year, and gave project staff increased time and control over recruitment efforts, retention activities, and intervention management while also controlling staffing costs. All participants provided email, address, and phone contact information. The enrollment goal was 210 parents (with half in each study condition), based on an *a priori* power analysis for analysis of covariance (controlling for baseline scores) calculated with G*Power software version 3.1.9.2 (Universitat Kiel, Germany), which was set for 2 groups with a small effect size (0.25), *P*-value (alpha)=.05, and power (beta)=0.95. A recruitment goal of approximately 300 parents was established to allow for attrition.

### Intervention Content

#### Experimental Group: Healthy HomeStyles

**Figure 2:**
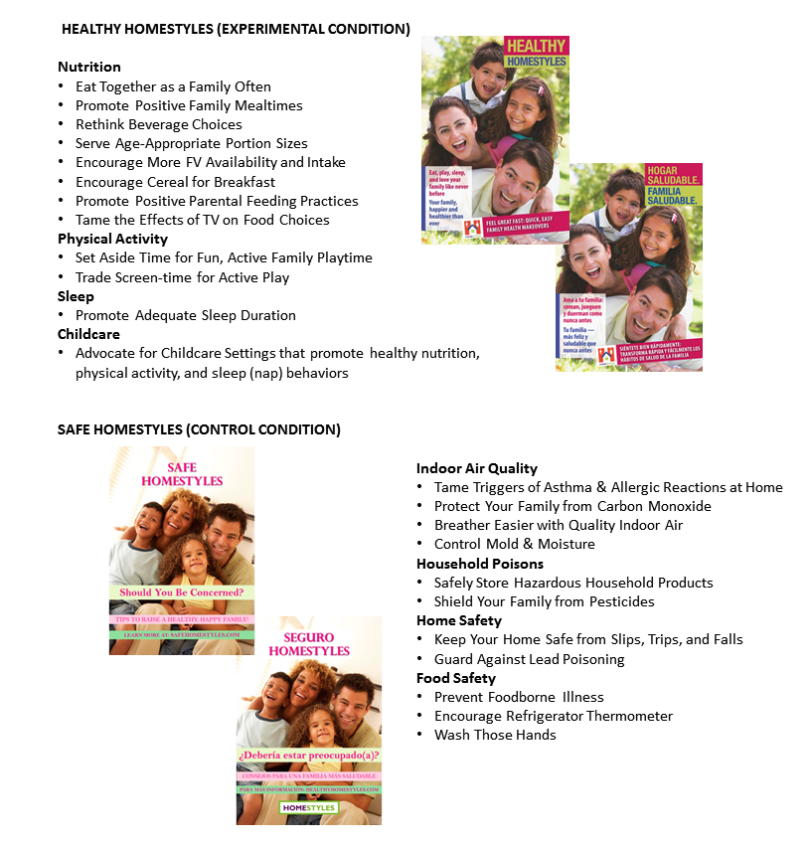
HomeStyles Key Messages.

The HomeStyles experimental group intervention materials (Healthy HomeStyles) are described in detail elsewhere [43,44]. In brief, the materials were delivered via the Web and consisted of 12 instructional guides in the form of 4-page mini-magazines. The introductory guide provided an overview of the project and was designed to help parents select the other guides that would be most useful to their families. Each of the other 11 guides focused on one key nutrition, physical activity, or sleep message (Figure 2). All guides included ideas to help parents advocate to their child care providers for healthy nutrition, physical activity, and sleep (nap) practices because the vast majority of preschoolers spend at least some time in nonparental childcare settings. Children cared for at home by their parents are less likely to be obese than children cared for by others, suggesting a need for a larger parental role in the management of their children when they are away from home [45-49].

##### Organization and Content of Intervention Materials

Each guide was organized similarly and content was created and ordered using motivational interviewing strategies [50,51]. The guide cover was a full-color photograph showing children alone or with parents engaging in an activity reflective of the guide content. Cover lines designed to capture parent attention were displayed on each cover [44,52]. The inside verso page briefly summarized the evidence-based research indicating why the guide’s topic is important for good health, reminded parents that they are their children’s most important role models, helped parents reflect on why the behaviors discussed in the guide are important to them personally using scaling (eg, “How important is this to you on a scale of 1 to 10? Why did you choose that number? What would have to be different for you to choose a higher number?”), and encouraged them to make simple changes to promote optimal child health. The inside recto page provided tips and ideas from other parents of preschoolers who previously participated in HomeStyles-related focus groups, offered more information on the guide’s topic, gave parents another opportunity to consider why the behaviors promoted in the guide were important to them, and encouraged them to make simple changes. The back of the guide focused on goal setting and provided examples of goals that other parents set, and encouraged parents to think about goals that they could set for their families. The guide concluded with a brief summary and reminder to choose small, manageable changes that are important to their families. Full-color photographs and a variety of graphic elements (eg, shading, highlighting, font treatments) were used to enhance the visual appeal of the guides. Each guide took approximately 15 minutes to review.

Each guide focusing on a key message was accompanied by a goal tracking sheet that encouraged parents to set goals related to the guide, monitor progress toward goals, and reward their family for reaching goals [53]. Most key message guides were accompanied by enhancements (objects with instructions/ideas for use) that facilitated implementation of the promoted behaviors. Examples of enhancements included: measuring cups for the portion sizes guide, a cutting board for the fruits and vegetables guide, a family mealtime conversation idea fan deck for the family meals guides, colorful straws and recipe ideas for the beverage guide, hula hoops and beach balls for the physical activity guides, and timers for the taming television guide. Enhancements were mailed to participants.

A series of *gentle nudges* was created and cognitively tested for all guides [54]. Nudges are periodic, brief communications that provide social support to help individuals change their behaviors [55,56]. Nudges were designed to be delivered by phone, text messaging, and/or email, per participant preference. A password-protected website was constructed to: deliver the guides, goal trackers, and nudges; enable participants to see how they were progressing through the intervention; and provide additional information (eg, how to get the most out of participation, set goals, build self-confidence, and cope with stress).

##### Intervention Development Process

The development process used for all experimental group intervention materials ensured inclusion of components of effective health interventions. Namely, processes were based on current obesity prevention guidelines and up-to-date research literature, had a strong theoretical basis (ie, Social Cognitive Theory [5,57]), used participatory planning and implementation strategies (ie, stakeholder [parents of preschoolers] input, advisory group, Adult Learning Theory [58-63]), and conveyed clear messages to participants (ie, designed using the Attention, Relevance, Confidence, Satisfaction [ARCS] Model of Motivational Design [58,59]) that were delivered using motivational interviewing principles [50,51,64,65].

Key obesity prevention guidelines used to develop HomeStyles were from the Institute of Medicine, Centers for Disease Control and Prevention, White House Task Force, Dietary Guidelines for Americans, and Healthy People 2020 [24,66-69]. Extensive literature reviews were conducted to ensure the most salient home environment and lifestyle practices were targeted in the intervention, and to inform the content of the intervention materials [70,71].

Bandura’s Social Cognitive Theory [57] provided the theoretical underpinnings for HomeStyles. This theory’s concept of *reciprocal determinism* was well suited to HomeStyles in that it expresses the idea that environment, behaviors, and personal characteristics mutually and concurrently affect each other, and that humans have the capacity to form or transform environments to support desired behavioral outcomes [5]. The behavior change strategies employed in intervention materials that aimed to develop and support this capacity for change included: outcome expectations, attitudes and values, self-efficacy, collective (family) efficacy, vicarious observational learning (learning about how other parents achieved child weight-protective goals), barrier removal/support building, and self-regulation (ie, goal setting, self-reward, and enlisting social support) [5,72,73].

Numerous participatory planning and implementation strategies were employed to ensure HomeStyles intervention materials were responsive to the wants and needs of the target audience. For instance, parents of preschoolers were involved throughout the development, implementation, and evaluation of all aspects of the intervention. Before intervention material development began, 139 parents participated in focus groups to elucidate their quality-of-life determinates and weight-related behaviors, views, aspirations, and obstacles [74]. During intervention material development, 512 parents participated in cognitive testing interviews to verify that intervention material content was useful, attention-catching, clear, appealing, relevant, interesting, motivating, warm and understanding in tone, and *guilt-free* [43]. The advisory group was consulted regularly throughout all phases of the intervention to ensure that the most current knowledge and best practices were employed. Additionally, Adult Learning Theory guided HomeStyles development. This theory recognizes that effective instruction for adult learners involves them as equal partners, respects their knowledge-base and life experiences, clarifies the importance of the content, addresses their desire for relevant and worthwhile content, and promotes rapid application of knowledge to meet their goals [58-63].

ARCS and motivational interviewing were used in tandem with Adult Learning Theory. ARCS focuses on motivating learners by capturing their *attention*, providing *relevant* content, and targeting learner *confidence* and *satisfaction* [58,59]. Motivational interviewing is a client-centered, goal-oriented counseling strategy that facilitates behavior change by helping individuals clarify goals and aspirations, explore and resolve ambivalence to changing behavior, build self-efficacy for behavior change, stimulate intrinsic motivation to change behavior, and make plans to change [50,51,64,65]. Although originally developed for in-person counseling sessions, motivational interviewing strategies can be successfully used in self-instructional written materials [75].

#### Control Group: Safe HomeStyles

An attention control group was used to promote participant retention and control for nonspecific treatment effects (eg, participant burden, activity and data collection format, study event scheduling, attention from researchers). Thus, the control group received a bona fide treatment equal in nonspecific treatment effects to, and contemporaneously with, the experimental group [76,77]. A total of 12 intervention guides were designed for the control group. Figure 2 lists the topic of each guide. As in the experimental condition, an introductory guide was developed to provide an overview of the project to help parents select subsequent guides that would be of greatest value to their families. Content for the remaining guides (Safe HomeStyles) was derived from government-produced home safety [78] and food safety and handwashing [79-85] educational materials, and reformatted into 4-page mini-magazines to have a look and feel similar that of the experimental condition. Additionally, nudges were written, enhancements were developed (eg, refrigerator thermometer, reminder magnets and wrist bands, lint-free dust cloths, stickers for labeling household poisons), and website content was created. Control group participants only had access to Safe HomeStyles materials on the website, whereas experimental group participants could only access the website material focused on Healthy HomeStyles.

### Randomized Controlled Trial Design

**Figure 3:**
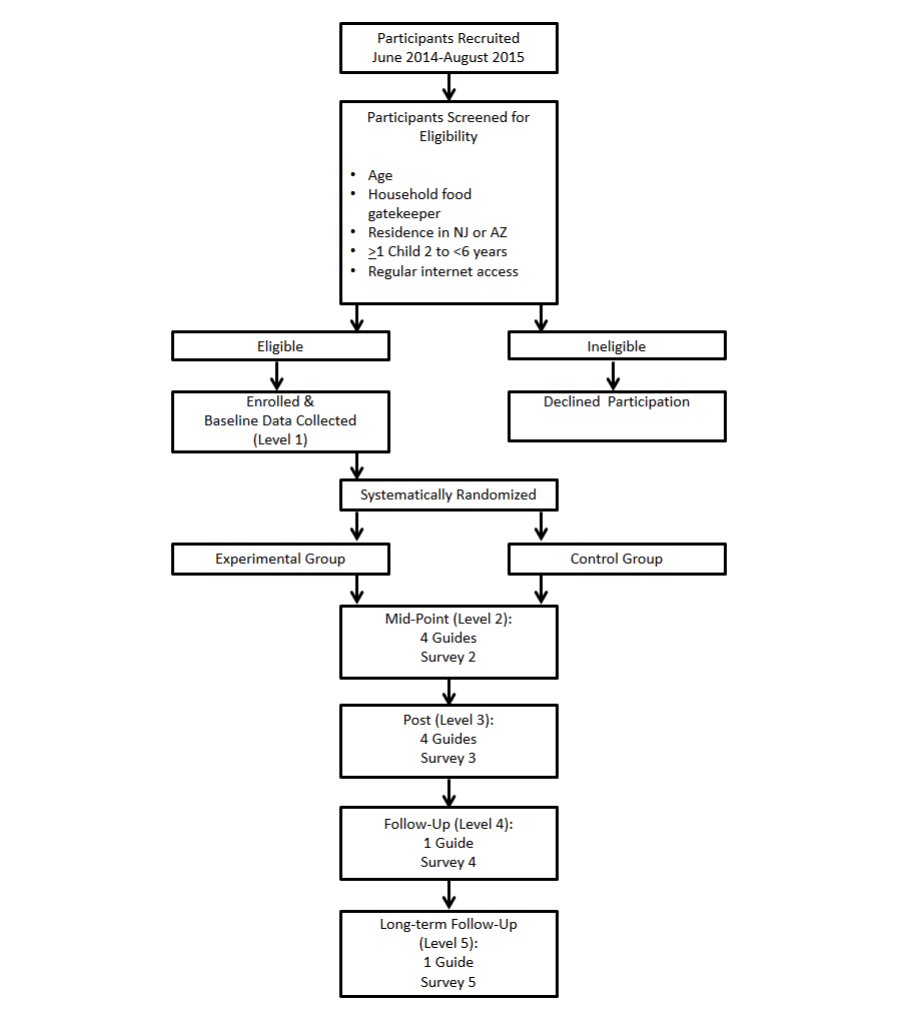
HomeStyles intervention study flow based on CONSORT guidelines.

Figure 3 illustrates the flow of the intervention study based on CONSORT guidelines [86]. Recruited participants began by completing a brief eligibility screener survey. Those who were eligible began the first of five sequential levels of participation. Each subsequent level of the intervention commenced immediately after concluding the activities in the previous level. In Level 1, participants completed the baseline survey online, after which they were systematically randomized by computer into the control or experimental group. Recruitment materials and the bona fide treatment delivered to the attention control group format were designed to blind participant assignment to study condition. In Level 2, participants received the introductory guide and were then able to select a new guide approximately every 16 to 30 days. After a total of 4 guides in Level 2, participants completed the second (mid-point) survey. In Level 3, participants selected 4 additional guides, with a new guide approximately every 16 to 30 days, then completed the third (post) survey. In Level 4, participants selected one new guide or revisited a previously selected guide, per their choice, and approximately 30 to 60 days later they completed the fourth (follow-up) survey. Similarly, in Level 5, participants selected one new guide or revisited a previously selected guide and completed the fifth (long-term follow-up) survey approximately 30 to 60 days later. Completion of all levels was designed to take approximately 12 to 18 months. Levels 1 to 3 were the main components of the intervention; Levels 4 and 5 were designed to allow assessment of longer-term intervention effects.

After selecting a guide, parents were encouraged to: spend approximately 15 minutes reviewing it; think about changes like those suggested in the guide that could help their families; select one or two simple, quick, low-cost changes to implement in their homes for a few weeks; and then choose a new guide. After selecting a guide on the website, parents were sent a hard copy of the guide and, for at least every second guide selected, they were sent an enhancement that supported the guide. Participants received a nudge approximately 5 days after selecting a new guide, and then every 5 days for 30 days they received a new nudge; during this period, the first 4 nudges were guide-specific and the final 2 were reminders to visit the website to choose a new guide.

Project staff closely monitored participant progress throughout the study by observing their visits to the website. To minimize attrition, participants that were not progressing through the study in the expected time frame received friendly reminders (by phone and email) that encouraged them to return to the website to complete the next activity (choose new guide or complete a survey). In addition, bilingual project staff were trained in customer service and rapidly responded to participant queries coming in via email or the project’s toll-free phone line using scripted responses to ensure similar and equal treatment across study groups. Participants also received modest, but increasing stipends after completing each survey. Other strategies to retain study participants included mailed enhancements, holiday cards, and opportunities to earn *bonus bucks* (an extra US $1) by visiting the website and answering a quick, fun question (eg, “Oprah’s Calling! What should she know about HomeStyles?”) that was refreshed approximately every 10 days [52].

### Instruments

#### Survey Development

Development of the study instruments began with an extensive literature search to identify the most salient sociodemographic characteristics and environmental, behavioral, and psychographic constructs that affect weight-related aspects of the home environment and lifestyle patterns. The literature review also served to identify valid, reliable, brief, and easy-to-administer self-report instruments suitable to the study purpose and target audience [87,88]. Furthermore, survey development involved a review of instruments that were previously developed by the HomeStyles researchers and used in analogous investigations [89]. When multiple scales for assessing a characteristic or construct were identified, a panel of nutrition experts in tests and measurements determined which was best suited to the study. For lengthy scales (ie, exceeding 6 items), published psychometric and factor analysis data were scrutinized to determine whether these scales could be shortened to reduce participant burden while preserving instrument integrity, validity, and reliability [90]. In the few cases where published psychometric data for lengthy scales could not be located, the panel of experts identified the most salient items.

When appropriate instruments could not be located, they were developed *de novo*. The process that was used to develop and refine scales followed Redding et al’s sequential approach to measurement of health behavior change constructs [91]. If scales contained items that were heavily modified from their original form or developed *de novo*, five experts in subject matter areas appropriate to the scale content (eg, nutrition, physical activity, psychology, child development, obesogenic environment), psychometrics, and survey design reviewed them to ensure scale clarity and content validity (ie, items in the scale reflect the characteristic being measured) [92,93]. The scales were iteratively reviewed by experts and refined until all experts agreed on scale content and construction. These scales were then subjected to cognitive testing by participants with characteristics similar to the study population (but did not participate in the final study) to ascertain whether participants interpreted the items as intended, and allowed us to identify ways to reduce participant burden and increase acceptability [92,94-96]. During cognitive testing, participants read each item aloud and then stated in their own words what the item was asking, answered the item, and explained their answers. At the end of the cognitive interviews, participants answered open-ended questions to determine how the items could be refined to make them easier to understand and faster (less burdensome/more acceptable) to complete. Items underwent iterative refinement and cognitive testing until they were clearly understood by, and acceptable to, the target audience [91]. The Home Opportunities for Physical activity check-Up (HOP-Up) scale is an example of the application and outcome of this scale development process [97].

All survey items were assembled into an online survey (using Qualtrics) that was pretested with 48 individuals with characteristics like those eligible for the final study (but did not participate in the study) to identify refinements needed to improve clarity, layout, reading ease, and acceptability, and to verify accuracy of scale scoring algorithms. A pilot-test with 550 individuals like those in the pretest identified a small number of additional refinements and confirmed usability, internal consistency, scale unidimensionality, and participant satisfaction [98]. As a final check, the panel of experts again reviewed the survey and pilot-test outcomes and confirmed the measures’ integrity and suitability to the study purpose.

#### Sociodemographic Appraisals

The survey included an array of sociodemographic assessments that permit the construction of a description of study participants. Data collected included parent age, sex, race/ethnicity, education level, and marital status as well as the *target* child’s age and sex. Parents having more than one preschool child were instructed to report on the child born closest to noon on June 1 (*a priori* randomly selected time and date). Parents rated their own and their child’s health status (poor, fair, good, very good, or excellent) using the Centers for Disease Control and Prevention’s Health-Related Quality of Life questionnaire [99,100]. Hager et al’s food insecurity screener was used to assess family food insecurity risk [101]. Family socioeconomic status was assessed with the 4-item Family Affluence Scale [102,103].

#### Outcome Measures

The outcome measures were organized according to the social ecological framework: individual (intrapersonal) assessments of parents and children, family/social interaction (interpersonal) evaluations, and home physical environment appraisals. As shown in Multimedia Appendix 1, primary outcome measures were home environment characteristics and lifestyle practices. Secondary outcome measures included parental behaviors, child behaviors, parental values, and self-efficacy for obesity preventive practices.

For items pertaining to children, parents were asked to answer them with their preschool children in mind. To keep parent attention directed to this *target* child, the child’s name (or nickname) entered in the survey by the parent was automatically populated in survey items pertaining to the child. For instance, “In the past week, how many days did *Tommy* run, jump...”, rather than “… how many days did *your child* run, jump…” Multimedia Appendix 1 organizes the outcome measures according to a social ecology framework and includes multiple details, including the number of items on scales, answer choice options and scoring, possible score range, references, and relationship to social cognitive theory constructs.

Individual measurements included height and weight data which were used to calculate the parent’s body mass index (BMI). Height, weight, age, and sex data were used to calculate the children’s BMI percentile [104]. To increase accuracy of height reports, participants were instructed to measure height using the special tape measure and instructions that were mailed to their homes, along with a brief video posted on the Internet [105].

Food frequency questionnaires determined parents’ daily servings of fruits/vegetables, milk, and sugar-sweetened beverages, and percent total calories from fat [106-111]. Physical activity levels were measured using the streamlined version of the International Physical Activity Questionnaire [112-114], which evaluated frequency of engaging in three levels of exercise (walking, moderate activity, heavy activity) during the past week. Parents also reported total daily time spent using sedentary screen-time devices (ie, watching television or DVDs, playing games on computers or smart phones) [98,115]. Pittsburgh Sleep Quality Index items evaluated usual daily sleep duration (hours/night) [116,117]. Using questionnaires analogous to those used with adults but appropriate for children, children’s daily servings of fruit and vegetable juice, milk, and sugar-sweetened beverages [106-111,118], physical activity level [112], total daily screentime [98,115], and daily total hours of sleep (night and naps) were assessed [116,117].

Family/social interactions that were measured included family mealtime and physical activity behaviors as well as factors affecting these interactions (ie, parental self-efficacy and values). Items determined family meal frequency [119] and locations [120-122], family mealtime environment characteristics [98,120,123], family meal planning [124-127] and self-efficacy [125], and parent modeling of healthy eating behaviors [30,128,129]. Scales also assessed how frequently parents played actively with their children [98], frequency of parental modeling of physical activity and sedentary behavior to children [114,120,123,128,130], and parental encouragement of children to be physically active [98,123,128,131,132].

Parental self-efficacy for promoting obesity-preventive behaviors in children [98,133,134] was assessed by having parents indicate how confident they felt in their abilities to keep children’s weight healthy and engage in obesity-preventive eating, physical activity, and sleeping behaviors. Parental values associated with achieving obesity-preventive home environments and lifestyles were assessed with scales measuring healthy eating outcome expectations (belief in link between diet and health) [125,135], physical activity outcome expectations (belief in link between exercise and health) [125,135], and values placed on modeling physical activity [98,123,130-132], not modeling sedentary behavior [98], and physical activity for children [131,132].

The home physical environment characteristics that were assessed included household food, physical activity, and media environment. Household food availability was assessed with food frequency-type questionnaires that yielded weekly servings typically available per person in the household consisting of: salty, fatty snacks; sugar-sweetened beverages; fruits and vegetables; breakfast foods; and milk [106,107,109,118,136]. The HOP-Up Checklist assessed physical activity availability and accessibility inside the home, in the area immediately outside the home/yard, and in the neighborhood [97]. The household media environment was described with items assessing the number and types of media devices (including television) in the home [120,123,128], the daily amount of time children were allowed to watch television/movies and use inactive media devices (eg, computers, tables, smart phones) [98], and total time each day that the television was on even if no one was watching [98,123].

### Data Management

At the outset of the project and continuously throughout the project, the project team established and refined data management procedures. These procedures included data file naming, processing, and storage conventions. Standard operating procedures were drafted and training modules were created. All staff involved in data collection and management were required to demonstrate competency in implementing the procedures before and during data collection or management activities. Supervisors regularly reviewed data collection and management procedures to ensure that they were followed with fidelity.

The data generated in this RCT project are primarily quantitative data gathered through surveys administered online. To ensure generated data are reliable, valid, and usable, the team strictly adhered to best practices for instrument development, online data collection, and psychometric analyses. Data generated via online survey software were downloaded in spreadsheet format at least twice weekly. Data were checked regularly to ensure accuracy of data capture. A comprehensive data dictionary that includes original items, answer choices, scoring/coding of answers, scoring of scales, and examples was created to ensure that all project data are accurately and readily usable.

To ensure the long-term preservation of the data generated in this study, during the execution of the project the research team store data files on password-protected university servers that are backed up daily. For added redundancy, all project data are shared periodically (at least twice weekly during active data collection periods) by key project staff on password-protected computers.

### Data Analyses

For all outcome measures, paired *t*-tests will be used to compare within-group differences over time, and analyses of covariance (controlling for baseline scores) will be used to determine differences in midpoint, post, follow-up, and long-term follow-up of online survey scores between control and experimental groups. Hierarchical linear modeling will be used to analyze data longitudinally and compare the slope of independent variables as they change over time, as well as mean response. An overall multiple regression model will be developed to examine the difference between baseline and subsequent administration of the measures. Data for study completers and noncompleters will be examined as well, to determine how participants differ.

## Results

Enrollment for this study was completed in August 2017 and statistical data analyses are underway as of March 2017.

## Discussion

The HomeStyles project was designed to help families with young children shape home environments and lifestyles to promote optimal child growth and development, and prevent childhood obesity using quick, easy, low-cost strategies that can become part of their everyday lives. Using electronic technology to deliver HomeStyles materials enables parents to access materials at times and locations most convenient to them. HomeStyles was based on current obesity prevention guidelines, a multifactorial (diet, physical activity, sleep) approach, participatory planning and implementation strategies, and conveyed clear, actionable messages delivered using motivational interviewing principles [50,51,64,65]. These approaches help to ensure that HomeStyles is responsive to the wants and needs of parents with preschoolers. These considerations also increase the potential of HomeStyles’ messages resonating with (and being acted on) by the target audience, thereby contributing to the long-term goal of improving health and preventing childhood obesity.

Extensive attention was devoted to overcoming challenges noted in previous studies. For example, parental reports of children’s heights and weights tend to be inaccurate [137-141]. Thus, to improve parent accuracy, HomeStyles researchers developed and validated a height measurement kit [105]. This kit included a special tape measure that labeled each quarter-inch increment, a plumb line made of a metal washer and mason’s line, and instructions (written and video) explaining how to hang the tape measure straight on a wall to accurately measure children. Additionally, the researchers verified that home bathroom scales provide sufficiently accurate and consistent weights for public health results [142].

All too often, the materials developed for interventions contain excellent content but poor design. Consumers have come to expect materials equivalent in quality to professional magazines and websites. The HomeStyles project avoided the *homemade* look by including a professional graphic artist who had extensive experience with both the target audience and subject matter, and was fully embedded in this project from conceptualization to execution. The resulting materials have a unified look and feel and were branded with the HomeStyles logo to promote *brand awareness*. A professional copyeditor ensured that all materials were grammatically correct and uniformly formatted.

Another challenge in the development of materials is offering them in more than one language. All HomeStyles materials were developed and fully tested in audiences whose primary language was either English or Spanish. Spanish translation is especially complicated because it is spoken in many countries, each of which has some unique phrasing and terms for common words (eg, the terms snack, lunch, and straws) that may be not be understandable in all countries. Further complicating this issue is the use of English words along with Spanish among immigrants to the United States. To address this translation challenge, HomeStyles engaged professional translators who were familiar with the subject matter and who used *in-culture* translations (ie, translations using terms and phrasing common to the vast majority of Spanish speakers). Another challenge of the Spanish translation in this project was to capture the warm, inviting, guilt-free, personalized tone that was used in the English materials. To achieve the desired tone, the professional translators were included in the development of project materials from the outset of the project, before even one word was written. In addition, many of our project staff were bilingual with varied nativity, including Chile, Bolivia, Mexico, and Puerto Rico.

Journal articles reporting nutrition RCT outcomes frequently indicate that the intervention was based on a particular behavior change theory [143,144]. However, there is rarely any indication of which constructs from the theory were used, or documentation as to where or how the theory was employed. To overcome this challenge and advance the field, the use of Social Cognitive Theory constructs and motivational interviewing strategies in the HomeStyles guides are the first (to our knowledge) to be clearly annotated. These annotations are described in detail elsewhere [43].

Recruiting study participants is a significant obstacle faced by researchers. Compounding this issue is, “a clear knowledge gap with regard to effective strategies aimed at those recruiting to trials” [145]. The HomeStyles study aimed to overcome recruitment obstacles in a variety of ways. For instance, the project used an attention control group that provided these participants a plausible, useful, bona fide treatment. An added benefit of this design is that it minimizes threats to internal validity [146-148]. The project also used intensive and varied recruitment strategies. Additionally, the project offered participants tangible incentives in the form of enhancements and stipends, as well as intangible incentives that parents indicated were important to them (eg, providing intervention materials that would help them build happier, closer bonds with their children). These incentives were used to help reduce participant attrition, which is another critical obstacle to the successful completion of intervention studies.

Effective childhood obesity prevention programs are desperately needed to reverse the obesity epidemic. To our knowledge, HomeStyles is among the first large-scale, rigorously controlled studies to test the effectiveness of a multifactorial childhood obesity prevention program that aims to help parents shape their home environments and lifestyle practices using quick, easy, low- or no-cost strategies. The thoughtfully developed, evidence-based, behaviorally focused, theory-driven content of the intervention materials is expected to enable and motivate parents to promote optimal home environments, lifestyle practices, child growth, health, and body weights.

## Appendix

Multimedia Appendix 1 HomeStyles study measures.

Multimedia Appendix 2 Peer review report for funded behavioral intervention study.

Multimedia Appendix 3 CONSORT checklist.

## Abbreviations

ARCS: Attention, Relevance, Confidence, Satisfaction
BMI: body mass index
HOP-Up: Home Opportunities for Physical activity check-Up
RCT: randomized controlled trial
