# Supplementary material for: HomeStyles, A Web-Based Childhood Obesity Prevention Program for Families With Preschool Children: Protocol for a Randomized Controlled Trial
Source: JMIR Res Protoc. 2017 Apr 25;6(4):e73. doi: 10.2196/resprot.7544 (PMC5424124; doi:10.2196/resprot.7544)
Supplement: Multimedia Appendix 1 [file resprot_v6i4e73_app1.pdf]

Multimedia Appendix 2. HomeStyles study measures.

Measures	# of Items	Scale Type	Possible Score Range
**Individual (Intrapersonal) Measures**			
Parents			
Health Status [99, 100]	1	5-point excellence rating [A]	1-5
BMI	1	BMI units	0-no limit
Behaviors [M]			
Fruit/Vegetable (servings/day) [108-111]	7	6-point servings eaten scale [B]	0-12.9
Milk (servings/day) [106-111, 118]	1	9-point servings drank scale [C]	0-8
Sugar-sweetened Beverages [106, 107] (servings/day)	4	9-point servings eaten scale [C]	0-4.6
Percent Total Calories from Fat [108-111]	17	5-point servings eaten scale [D]	0-100
Physical Activity Level [112-114]	3	8-point exercise scale [E]	0-42
Screentime [98, 115] (minutes/day)	1	time/day	0-1440
Sleep Duration (minutes/day) [116, 117]	1	time/day	0-1440
Children			
Health Status [99, 100]	1	5-point excellence rating [A]	1-5
BMI percentile [104]	1	percentile	0-100
Behaviors [M]			
Fruit/Vegetable Juice (servings/day) [106-111, 118]	2	9-point servings drank scale [C]	0-2.3
Milk (servings/day) [106-111, 118]	1	9-point servings drank scale [C]	0-8
Sugar-sweetened Beverage (servings/day) [106-111, 118]	2	9-point servings drank scale [C]	0-2.3
Physical Activity Level [112]	3	8-point Exercise scale [E]	0-42
Screentime minutes/day [98, 115]	1	minutes	0-1440
Sleep Duration (minutes/day) [116, 117]	1	minutes	0-1440
**Family/Social Interactions (Interpersonal) Measures**			
Food-Related Lifestyle Practices [L]			
Family Meal frequency/week [119]	3	0-7 days for breakfast, lunch, dinner; score is sum of 3 meals	0-21
Family Meal Location [120-122]			
In Car (days/week)	1	0-7 days	0-7
At Fast Food Restaurant (days/week)	1	0-7 days	0-7
At Dining Table (days/week)	1	0-7 days	0-7
In Front of TV (days/week)	1	0-7 days	0-7
Media Device Use at Family Meals [98,	1	0-7 days	0-7

120, 123] (days/week)			
TV Use at Family Meals & Snacking Occasions [98, 120, 123] (days/week)	1	0-7 days	0-7
Family Mealtime Emotional Environment [122, 123]	2	5-point agreement rating [F]	1-5
Family Meals are Planned [124-127]	2	5-point agreement rating [F]	1-5
Parent Family Meal Preparation Self-Efficacy [125]	2	5-point agreement rating [F]	1-5
Parent Modeling of Healthy Eating [30, 128, 129]	4	5-point agreement rating [F]	1-5
**Parent Self-efficacy for Food-Related Childhood Obesity-Preventive Practices** [M] [98, 133, 134]			
Self-efficacy for Keeping Child's Weight Healthy	1	5-point confidence rating [G]	1-5
Self-efficacy for Letting Child Decide Amounts to Eat at Mealtime	1	5-point confidence rating [G]	1-5
Self-efficacy for Getting Child to Eat Breakfast	1	5-point confidence rating [G]	1-5
Self-efficacy for Getting Child to Eat Fruits & Vegetables	1	5-point confidence rating [G]	1-5
Self-efficacy for Getting Child to Drink Fewer Sugary Drinks	1	5-point confidence rating [G]	1-5
Self-efficacy for Separating TV Watching and Eating	1	5-point confidence rating [G]	1-5
Physical Activity-Related Lifestyle Practices [L]			
Parent: Child Co-Physical Activity (days/week) [98]	2	8-point modeling scale [H]	0-7
Parent Modeling of Physical Activity (days/week) [114, 120, 123, 128, 130]	2	8-point modeling scale [H]	0-7
Parent Modeling of Sedentary Activity (days/week) [114, 120, 123, 128, 130]	2	8-point modeling scale [H]	0-7
Parent Encouragement of Child Physical Activity [98, 123, 128, 131, 132]	5	5-point agreement rating [F]	1-5
Parent Self-Efficacy for Physical-Activity Related Childhood Obesity-Preventive Practices [M] [98, 133, 134]			
Self-efficacy for Getting Child to Be Physically Active	1	5-point confidence rating [G]	1-5
Self-efficacy for Getting Child to Limit Screentime	1	5-point confidence rating [G]	1-5

Self-efficacy for Getting Child Enough Sleep	1	5-point confidence rating [G]	1-5
Parent Values Related to Obesity-Preventive Practices [M]			
Healthy Eating Outcome Expectations [125, 135]	6	5-point agreement rating [F]	1-5
Physical Activity Outcome Expectations [125, 135]	6	5-point agreement rating [F]	
Value Placed on Modeling Physical Activity [98, 123, 130-132]	2	5-point agreement rating [F]	1-5
Valued Placed on Not Modeling Sedentary Behavior [98]	1	5-point agreement rating [F]	1-5
Value Placed on Physical Activity for Children [131, 132]	2	5-point agreement rating [F]	1-5
**Home Physical Environment Measures** [L]			
Household Food Availability [106, 107, 109, 118, 136]			
Fruits/Vegetables (servings/household member/week)	7	9-point servings scale [I]	†
Salty/fatty snacks (servings/household member/week)	4	9-point servings scale [I]	0-32
Sugar-sweetened Beverages (servings/household member/week)	4	9-point servings scale [I]	0-32
Breakfast Foods (servings/household member/week)	3	9-point servings scale [I]	0-24
Milk (servings/household member/week)	1	9-point servings scale [I]	0-8
Physical Activity Environment [L] [97]			
Indoor Home Space & Supports For Physical Activity	6	Varies by item; 2 items are counts; 1 item is a 5-point agreement rating [F]; 3 items are 5-point occurrence ratings [J]	1-5
Outdoor/Yard Space & Supports For Physical Activity	4	5-point agreement rating [F]	1-5
Neighborhood Space & Supports For Physical Activity	4	5-point agreement rating [F]	1-5
Neighborhood Environment Safety	2	5-point agreement rating [F]	1-5
Frequency of Active Play Outdoors	2	5-point occurrence ratings [J]	1-5
Media Environment [L]			
Total Number of Inactive Media Devices (including TV) in the Home [120, 123, 128]	6	Total devices[K]	0-66

Time Children are Allowed to Watch TV/Movies & Use Inactive Media Devices (e.g., computers, tablets, smart phones) [98] (minutes/day)	1	minutes	0-1440
Total Time TV is on When No One is Watching [98, 123] (minutes/day)	1	minutes	0-1440

[A] 5-point Excellence Rating: poor, fair, good, very good, excellent; scored 1 to 5 respectively; higher score indicates better health.

[B] 6-point Fruit/Vegetable Servings Rating: <1 serving/week, 1 serving /week, 2 to 3 servings/week, 4 to 6 servings/week,1 serving/day, 2 or more servings/day; scored 0 to 5 respectively; scale scoring algorithm is protected by copyright and described in detail elsewhere [109, 110].

[C] 9-point Sugar-sweetened Beverage Servings Rating: <1 time/week, 1 day/week, 2 days/week, 3 days/week, 4 days/week, 5 days/week, 6 days/week, 7 days/week, >1 time/day; scored 0 to 8 respectively; daily sugar intake from these beverages is item score multiplied by grams of sugar in a typical serving divided 7 days/week [106, 107].

[D] 5-point Fatty Food Servings Rating: 1 time/month or less, 2 to 3 times/month, 1 to 2 times/week, 3 to 4 times/week, 5 or more times/week; scored 0 to 4 respectively; scale scoring algorithm is protected by copyright and described in detail elsewhere [109, 137].

[E] 8-point Exercise Days/week: 0, 1, 2, 3, 4, 5, 6, and 7; days/week weighted by exercise intensity (weights of 1, 2, 3 for walking, moderate, and vigorous activity, respectively) and summed to create scale score; higher scale score indicates greater activity level.

[F] 5-point Agreement Rating: strongly disagree, disagree, neither agree nor disagree, agree, strongly agree; scored 1 to 5 respectively with scoring reversed for negatively worded statements; scale score equals average of item scores; higher scale score indicates greater expression of the trait.

[G] 5-point Confidence Rating: not at all confident, not confident, confident, quite confident, very confident; scored 1 to 5 respectively; higher scale score indicates greater confidence.

[H] 8-point Modeling Days/week: 0 (almost never), 1, 2, 3, 4, 5, 6, and 7; days averaged to create scale score; higher score indicates more frequent modeling.

[I] 9-point Household Servings Rating: <1 time/week, 1 day/week, 2 days/week, 3 days/week, 4 days/week, 5 days/week, 6 days/week, 7 days/week, >1 time/day; scored 0 to 8 respectively.

[J] 5-point Occurrence Rating: almost never, 1-2 times/week; 3 to 4 times/week, 5 to 6 times/week, every day; scored 1 to 5 respectively; scale score equals average of item scores; higher scale score indicates greater occurrence of behavior.

[K] 11-point frequency scale for each media device: 1=1 to 10=10, 11=more than 10.

[L] Primary outcome measures.

[M] Secondary outcome measures.
