# Supplementary material for: HomeStyles, A Web-Based Childhood Obesity Prevention Program for Families With Preschool Children: Protocol for a Randomized Controlled Trial
Source: JMIR Res Protoc. 2017 Apr 25;6(4):e73. doi: 10.2196/resprot.7544 (PMC5424124; doi:10.2196/resprot.7544)
Supplement: Multimedia Appendix 2 [file resprot_v6i4e73_app2.pdf]

United States Department of Agriculture
National Institute of Food and Agriculture
Agriculture and Food Research Initiative
Childhood Obesity Prevention: Integrated Research, Education, and Extension to Prevent Childhood Obesity

Proposal 2010-04646 submitted by Byrd-Bredbenner

Homestyles: Shaping Home Environments And Lifestyle Practices To Prevent Childhood Obesity: A Randomized Control Trial

The review panel grouped proposals into one of the relative categories below. The percentage indicates the final distribution of proposals in each category.

Recommended for Funding:

Outstanding %	14
High Priority %	18
Medium Priority %	29

Not Recommended for Funding:

Low Priority %	7
Do Not Fund %	32

This proposal was placed in : Outstanding
===
Childhood Obesity Prevention: Integrated Research, Education, and Extension to Prevent Childhood Obesity - PANEL SUMMARY

The panel decision regarding your proposal is based on the input provided by the reviews and the collected expertise and judgment of the individual panel members. This panel summary reflects the consensus opinion of the panel regarding your proposal.

Proposal Number: 2010-04646   Project Director: Byrd-Bredbenner

Proposal Title: Homestyles: Shaping Home Environments And Lifestyle Practices To Prevent Childhood Obesity: A Randomized Control Trial

Objectives of the Proposal:
1. Determine whether an in-home intervention enables and motivates parents in New Jersey and Arizona to shape home environment and lifestyle practices.

2. Determine whether the effectiveness of the intervention is affected by program delivery mode (in-home, face-to-face vs independent learning online delivery mode).

Positive Aspects of the Proposal:
The integrated, multidisciplinary project has a solid research foundation. The focus on family and home environment would help fill research gaps that are critical to the development of practices and programs. This proposed project is a unique comprehensive approach to childhood obesity prevention that demonstrates nearly perfect alignment with the overall program area priority and requirements. It involves multiple institutions, multiple states (in different regions of the country), and multiple disciplines. The approach is theory based, innovative and multifactorial focusing on the preschoolers and the home environment, lifestyle practices including diet, exercise and sleep. Sleep is a unique focus of the intervention but well supported by the proposal. The project is fully integrated with clear statements of how it fits within the program area and the research, education and extension are clearly reflected in the objectives. It laid out a detailed timeline and comprehensive plan of work including workshops, trainings, and other field activities. The extension activities proposed could definitely lead to documented changes in learning, actions, or conditions. An English and Spanish version of print materials, on-line interventions and reference manual are particularly strong points. This would assist in helping LGU¿s and other agencies ensure that they are utilizing the most current, relevant, research-based information to reach diverse audiences. Key personnel appear to have sufficient expertise to complete the proposed project, and where appropriate, partnerships with other disciplines are established. The home visitors can help address the attrition rate as they typically visit participants starting prenatally and continue until the child enters preschool. The PD articulated a clear plan for project management, including time allocated for attainment of objectives and delivery of products, maintenance of partnerships and collaborations, and a strategy to enhance communication, data sharing, and reporting among members of the project team.
Negative Aspects of the Proposal:
Food frequencies may not be sufficient to detect subtle changes in intake as a result of an intervention and usually do not yield the best estimates of energy intake. Also, the intervention may bias participant responses towards more favorable responses rather than true changes in dietary intake.
Synthesis Comments:
The reviewers were excited about the potential of this project and its implications. It is not clear that the approach adequately builds on the body of literature and experience with similar programs. However, we believe that it will contribute greatly to the literature and Extension programs.
